# Transcranial Direct Current Stimulation Over the Right Anterior Temporal Lobe Does Not Modulate False Recognition

**DOI:** 10.3389/fpsyg.2021.718118

**Published:** 2021-09-17

**Authors:** María Angeles Alonso, Antonio M. Díez-Álamo, Carlos J. Gómez-Ariza, Emiliano Díez, Angel Fernandez

**Affiliations:** ^1^Instituto Universitario de Neurociencia (IUNE), University of La Laguna, San Cristóbal de La Laguna, Spain; ^2^Institute on Community Integration (INICO), University of Salamanca, Salamanca, Spain; ^3^Department of Psychology, University of Jaén, Jaén, Spain

**Keywords:** false memory, DRM paradigm, right anterior temporal lobe, semantic memory, brain stimulation

## Abstract

Non-invasive transcranial direct current stimulation (tDCS) over the left anterior temporal lobe (ATL) has been shown to cause a reduction in the rate of false memories with semantically related words. Such a reduction seems to be specific to false memories induced by the study of associative lists, but is not observed when the studied lists are categorical in nature. These findings are interpreted as evidence that the left ATL functions as an integration hub that is crucial for the binding of semantic information into coherent representations of concepts. In order to investigate whether the right ATL might also contribute to semantic integration in the processing of verbal associative material, a follow-up tDCS study was conducted with the stimulation at study lateralized on the right ATL. A sample of 75 undergraduate students participated in an experiment in which they studied 8 associative lists and 8 categorical lists. One third of the participants studied all their word lists under anodal stimulation, another third studied under cathodal stimulation and the other third under sham stimulation. Results showed that stimulation of the right ATL by tDCS does not modulate false recognition for either association-related critical words or category-related critical words. These results provide preliminary support to views positing asymmetric connectivity between the anterior temporal lobes and the semantic representational network, and provide evidence for understanding bilateral brain dynamics and the nature of semantically induced memory distortions.

## Introduction

Research on memory distortions using the Deese, Roediger-McDermott (DRM) paradigm (Deese, 1959; Roediger and McDermott, 1995) has consistently shown that presenting a list of words associated with a critical word not presented for study produces high levels of false recall and false recognition of that critical word (Gallo, 2006, 2010). There is strong evidence of a relationship between the memory illusion typically obtained with the DRM paradigm and aspects of semantic representation and processing (Gallo, 2010; Roediger and Gallo, 2016). A number of studies have demonstrated that this kind of memory distortions in list-learning experiments is critically modulated by the nature of lexical, semantic or structural similarity between to-be-remembered items and their related critical words (see Coane et al., 2021 for a recent review). And it has also been shown that many experimental manipulations that favor the processing of semantic characteristics of the studied words (e.g., meaning-oriented processing, relational processing, presentation of the material in meaning-consistent blocks, etc.) can cause an increase in false memories (Tussing and Greene, 1997; Thapar and McDermott, 2001; McCabe et al., 2004). Convergently, patient studies describe false memory effects that are modulated by the involvement of damaged semantic brain networks. As an example, patients with semantic dementia or fronto-temporal dementia, characterized by damage to the anterior temporal lobe (ATL), tend to show a reduction in false memories with DRM or similar tasks that require the construction of the general meaning or representation of the “gist” that summarizes the semantic characteristics common to studied list items (Simons et al., 2005; de Boysson et al., 2011).

In line with these findings, experiments with neuroimaging and non-invasive brain stimulation techniques in brain-intact participants have also shown the involvement of the ATL in the formation and modulation of false memory production with the DRM paradigm. Using fMRI, Chadwick et al. (2016) showed that the pattern of activation in the ATL while reading DRM lists predicted false recognition of the critical words associated to those lists. Going beyond correlational evidence, Gallate et al. (2009) found that altering the normal functioning of the left ATL using low-frequency repetitive transcranial magnetic stimulation (rTMS) reduced the probability of false recognition of the critical word without altering correct recognition. Consistently, Boggio et al. (2009) found a similar decrease in false recognition with anodal stimulation using transcranial direct current stimulation (tDCS) on the ATL, again with no stimulation effects on veridical memories. And more recently, Díez et al. (2017) found that the involvement of the ATL in this type of memory illusions depended on the kind of semantic relationship between the words in the list and the unstudied critical word. In their study, they applied transcranial direct current stimulation (anodal/cathodal/sham) in the left ATL and manipulated the type of semantic relationship (associative vs. categorical) between the words in the list and the critical items. The results of this study showed a significant reduction in false recognition with anodal stimulation in the left ATL, but only for those lists that had an associative relationship with the corresponding critical word. Although including only a small subset of the available evidence, the previous examples implicate that associative false memories are byproducts of relatively high-order semantic processes and that the ATL is a critical brain area for the representation of conceptual meaning.

One way in which the involvement of the ATL in the production of false memories can be more thoroughly understood is proposed by the “hub-and-spoke” model, a theoretical view that assumes that experiences (verbal and non-verbal) provide the basis for the formation of concepts and that this source of information is encoded in modality-specific areas distributed throughout the brain (the spokes). The model also assumes intermodal interactions for that specific information, mediated by a transmodal hub located in the ATL (Patterson et al., 2007; Lambon Ralph and Patterson, 2008; Lambon Ralph et al., 2010; Binney and Lambon Ralph, 2015; Patterson and Lambon Ralph, 2016). The model has more recently been enriched by the addition of proposal for a semantic control network and its brain correlates (Lambon Ralph et al., 2017; Chiou et al., 2018). In this framework, the anterior temporal region of both hemispheres would function as an integration hub, specialized in integrating modality-specific information from distributed brain areas to form coherent conceptual representations (Wong and Gallate, 2012; Bonner and Price, 2013; Lambon Ralph, 2014; Lambon Ralph et al., 2017).

The hub-and-spoke model, as developed to this moment, has been rather successful in accounting for a wide range of empirical findings, involving both healthy participants and brain-compromised patients. And it has also been formally validated in computational simulations (Hoffman et al., 2018). However, further evidence-based specification is needed regarding some particular aspects, such as the extent to which structures and networks in both sides of the brain play equivalent roles in the representation of semantic cognition. And along these lines, a question remains as to whether the left and the right ATLs have the same representational functions or contribute similarly to conceptual processing. There is sufficient clinical and experimental evidence to support a bilateral involvement of the ATL in semantic processing. What is not so clear, however, is whether both structures are as symmetric in terms of semantic processing as initially assumed. An alternative position is that there is hemispheric specialization of the ATL, with the left side specialized in verbal semantic representation and the right side specializing in non-verbal semantic representation (Gainotti, 2011, 2012). Indeed, data from several studies suggest that semantic impairment could be modality-specific in the early stages of the disease, with significant asymmetries between the left and right ATLs. In these cases, a more atrophic left ATL tends to have effects on lexical-semantic knowledge, while an atrophy in the right ATL tends to affect pictorial representations (Snowden et al., 2004). The hypothesis that the ATL in both hemispheres is asymmetric in terms of semantic processing is also supported by the conclusions of a large meta-analysis of neuroimaging studies (Rice et al., 2015b), with the data pointing toward a more lateralized left ATL involvement in semantic tasks that required the processing of verbal stimuli (Rice et al., 2015a).

In an attempt to provide further evidence, the present study examined the role played by the right ATL in the conceptual processing manifested in the production of false memories upon studying word lists of semantically related items. Such memory distortions are, in large part, a consequence of higher-order semantic processing, the kind of processing in which the ATL is purportedly involved. As mentioned above, this has been shown in prior studies in which modulating neural activity in the left ATL via non-invasive stimulation caused a reduction of false recognition (Boggio et al., 2009; Gallate et al., 2009), with the reduction particularly affecting false recognition of items that had an associative relationship with the studied material (Díez et al., 2017). Following this rationale, in the present study we aimed to modulate activity in the right ATL by using tDCS.

tDCS involves the delivery of a low-level intensity current by a battery-driven stimulator. The conventional procedure requires two electrodes (anode and cathode) with at least one of them being placed on the scalp. The current passes from anode to cathode and this current has been shown to modulate the neurons' electrical activity. While this current is not sufficient to induce action potentials, research has revealed that tDCS may change the response threshold of the reached neurons (Bindman et al., 1964; Brunoni et al., 2011). Specifically, and based on findings derived from research that mainly focused on motor cortices (i.e., Nitsche and Paulus, 2001), it is usually stated that anodal tDCS increases neuronal excitability (by depolarization), and that cathodal tDCS decreases neuronal excitability (by hyperpolarization) (Nitsche and Paulus, 2000; Cambiaghi et al., 2010). Hence, and because anodal stimulation is sometimes associated with enhanced performance (i.e., Cerruti and Schlaug, 2009; Tanaka et al., 2009) and cathodal stimulation is sometimes associated with worse performance (Stagg et al., 2011; Young et al., 2013), it is frequently stated that anodal tDCS leads to facilitate brain functions whereas cathodal tDCS disrupts them (see Fertonani and Miniussi, 2017 for a critical view). However, and when these polarity-dependent effects are frequently reported, evidence accumulates to show that such effects are far from being straightforward both at the neurophysiological (i.e., Antal et al., 2007; Tanaka et al., 2020) or the behavioral level (i.e., Gómez-Ariza et al., 2017; Friedrich and Beste, 2018), with anodal tDCS sometimes giving rise to performance that is compatible with the disruption of brain functions (i.e., King et al., 2020) and cathodal tDCS sometimes producing enhanced performance (i.e., Brückner and Kammer, 2017). Despite this, and when the specific action mechanisms underlying the possible behavioral effects of tDCS in humans remain largely unknown and are thought to depend on a number of factors (i.e., brain activity prior to stimulation, current intensity, targeted brain area/network), tDCS is considered a useful technique to better understand the neural substrates of cognition (Berryhill et al., 2014; Filmer et al., 2014; Bestmann et al., 2015).

The number of tDCS studies on (long-term) memory has increased over the years, even though the variability of stimulation protocols (i.e., electrode montages, duration…), goals (i.e., applied vs. basic research), employed memory tasks (i.e., associative vs. item memory) and memory-related processes (i.e., encoding vs. retrieval) is considerable (for a systematic review and meta-analysis on episodic memory, see Galli et al., 2019). Many of these studies seek to enhance performance by stimulating specific brain areas/networks thought to play a pivotal role in either encoding or retrieval processes. In other cases, studies using tDCS aim to test theoretically-guided hypothesis on the involvement of certain brain regions in memory processes or representations. Thus, for example, Leach et al. (2019) showed, in younger adults, that anodal stimulation over the left dorsolateral prefrontal cortex during a face-name encoding task improved associative memory. And Bjekić et al. (2019) found that anodal tDCS over either the left or the right posterior parietal cortex led to better performance on two different associative memory tasks. Interestingly, tDCS has also been used to dissociate the role of distinct memory-related brain areas. Pisoni et al. (2015), for example, showed that while tDCS over the left temporal cortex modulated recognition for studied items, stimulation over the right parietal cortex allowed participants to better identify new items (for other examples of dissociations see Pergolizzi and Chua, 2016, or Smirni et al., 2015).

Of special relevance here, tDCS has now been shown to be effective in modulating neural activity associated with representational aspects of semantic processing, sometimes contributing to hemispheric dissociations. Thus, for example, relative to sham or cathodal stimulation, anodal tDCS of the posterior superior temporal gyrus, which subsumes Wernicke's area, has been shown to lead participants to come up with associates that are more representative of the basic-level category of a presented image that worked as a cue. Similarly, anodal tDCS over the same temporal subregion was found to speed up the identification of meaningful word pairs, but not non-meaningful ones (Price et al., 2016). Interestingly, stimulation of the homologous region in the right hemisphere made participants faster at judging whether two words were semantically related by a subordinate meaning (Peretz and Lavidor, 2013). Hence, it would seem that the effects that tDCS over the temporal lobe have on semantic associations are hemisphere specific. Stimulation of the right hemisphere would seem to modulate semantic processing of subordinate and indirect associations, whereas tDCS of the left temporal lobe would modulate more semantically related concepts. Moreover, some tDCS studies have revealed laterality-dependent memory improvements, with memory for visuospatial information being modulated with right temporoparietal stimulation and memory for verbal information being modulated with left temporoparietal tDCS (i.e., Fiori et al., 2017; Antonenko et al., 2018).

Hence, we aimed to explore if the right ATL has an equivalent role to its left homologous in the production of semantic-based memory errors by modulating its neural activity via tDCS. If this was the case, tDCS over the right ATL should result in changes in the production of false memories, particularly of the associative kind. In order to test this prediction, and closely following the design employed by Díez et al. (2017), tDCS (both anodal and cathodal) was delivered over the right ATL to evaluate its effects on false memory with the DRM paradigm, using lists of words that maintained either associative or categorical relationships with their unstudied critical words. If the ATL of the two hemispheres had the same functionality in terms of semantic processing, tDCS of the right ATL should lead to a reduction in false recognition of associative critical words, without affecting either true recognition or false recognition in categorical lists. If, on the other hand, the functionality of the ATL is not equivalent in the two hemispheres, such a pattern of results should not be found following anodal stimulation of the right ATL. Because of the scarcity of studies combining DRM and non-invasive stimulation, and also because of our limited current knowledge on the neurophysiological effects of tDCS when applied outside sensory/motor cortices (see above), we were reluctant to make specific a priori predictions regarding type of lists and polarity effects for the case that the right ATL were actually different in semantic functionality from the left ATL. However, the results of the experiment could still be relevant to further understand bilateral brain dynamics and the nature of semantically-driven memory distortions. With this last goal in mind, the design of the experiment was not only aimed toward a standard quantitative assessment of true and false memory performance in the different stimulation conditions, but it was also supplemented to characterize the subjective feelings of recollection and familiarity in their recognition responses and their possible dependence on the role played by the right ATL. To this end, the remember/know (R/K) testing paradigm originally devised by Tulving (1985) was implemented and included in the final memory test. In a study by Pergolizzi and Chua (2015), bilateral tDCS on the parietal cortex failed to show any effect on R/K responses, but whether subjective determinants of recognition are to some degree affected by stimulation-induced changes in the functioning of the ATL is an unexplored question.

## Methods

### Participants

A total of 78 undergraduates were recruited from the student population at the University of La Laguna, Spain. All were native speakers of Spanish, with normal o corrected-to-normal vision, and they all gave written informed consent for their participation in the study. They received course credit as a basic compensation. Their ages ranged from 18 to 30 years (*M* = 19.98; *SD* = 2.72), and 82% were female.

The sample size was determined in advance to be at least the double of the most similar tDCS studies with significant reported effects (e.g., Boggio et al., 2009; *N* = 10 subjects by stimulation condition) and also considering the range of those used in standard DRM experiments, and the results of a power analysis performed with G*Power (Faul et al., 2007). We estimated the sample size to obtain an effect size similar to that obtained in the Díez et al. (2017) paper for the stimulation group X list type interaction (${\text{η}}_{\text{p}}^{2}=$ 0.10, *F* effect size of 0.33, i.e., a medium effect size) in a repeated measures within-between interaction (2 -categorical and associative- and 3 -stimulation groups-, respectively) and the results revealed that to achieve 0.80 power we needed a minimal sample size of 27 subjects.

All participants were right-handed, according to the Edinburgh Handedness Inventory (Oldfield, 1971), and they were randomly assigned to one of three experimental conditions. Following standard procedures in experiments involving tDCS, the participants were screened and excluded if they reported any psychiatric, psychological or neurological disorder or if they reported brain injuries, migraines, epileptic seizures or family history of epilepsy. The institutional ethical committee of the University of La Laguna approved the protocol, and the study was conducted in compliance with the Declaration of Helsinki (World Medical Association, 2013). Data from three participants were excluded from the analysis for failing to meet an accuracy criterion set for the recognition task^1^. The results obtained using the data from the remaining 75 participants (25 in each stimulation condition: anodal, cathodal and sham; 20, 22, and 22 females, respectively) are presented in this report.

### tDCS

Stimulation was delivered by a battery-driven electrical stimulator (TCT Research Ltd.) with an intensity of 2 mA. Following the stimulation protocol used by Díez et al. (2017), the current was transferred by two 5 × 7 cm rubber electrodes covered with saline-soaked sponges. For anodal stimulation, the anode was placed over site FT10 (BA 38/20), according to the International 10-10 System for EEG electrode placement, and the cathode was placed on the contralateral shoulder. For the cathodal stimulation, the cathode was placed over site FT10 and the anode was placed over the contralateral shoulder. Site FT10 is considered the closest electrode location to the right ATL (Acharya et al., 2016). Stimulation was applied for 20 min in both the anodal and cathodal conditions, using 10-s fade in/out ramps. For the sham stimulation condition, the electrodes were placed in the same positions as in the active stimulation conditions, with current ceasing to be applied after 60 s of stimulation. All participants completed the session without major complains or discomfort.

### Stimuli

The stimuli were the same previously used to successfully induce false memories in the tDCS study by Díez et al. (2017) and consisted of 24 critical words (CW) each related with two separate word lists: one associative list and one categorical list. The associative list was constructed selecting the first 10 associates of the CW on the basis of their backward associative strength (BAS), obtained from Spanish free-association norms (Fernandez et al., 2004, 2014). The categorical list was built selecting 10 words belonging to the same category as the CW, according to normative data in Spanish (Marful et al., 2015). Thus, for the CW *book*, the associative list consisted of the words *author, foreword, chapter, page, volume, edition, reading, read, epilogue, and reader*. For the same CW, the words in the categorical list were *magazine, newspaper, novel, encyclopedia, article, story, comic, notes, notebook, and dictionary*.

Sixteen word lists (8 associative and 8 categorical) were presented to each subject. The remaining 8 CWs and their corresponding lists served as control CWs and distractors in the recognition test. A counterbalanced assignment of lists to subjects was used to ensure that all word lists were displayed in all the different study conditions.

### Procedure

The experiment took place in a quiet laboratory with only one participant per session. First, participants filled out a personal data sheet and a screening questionnaire about medical and psychological conditions, and they also signed an informed consent form. As in Díez et al. (2017), after electrode placement, and coincident with the time in which the stimulation was started, participants were asked to perform a pen and paper visual-search task for an idle time of 7 min, consisting in circling with a pen the letters “n,” “p,” and “c” in words of a text written in an unfamiliar language. We decided to have participants engage in a specific task to minimize variability in brain/cognitive activity during stimulation. Because the encoding phase lasted about 8.5 min, and taking into account the time needed for reading the instructions, stimulation (20 min) started before encoding. This type of stimulation (partially offline partly online, in this case online during encoding) tends to show larger effects than entirely offline before encoding (see Galli et al., 2019).

**Figure 1:**
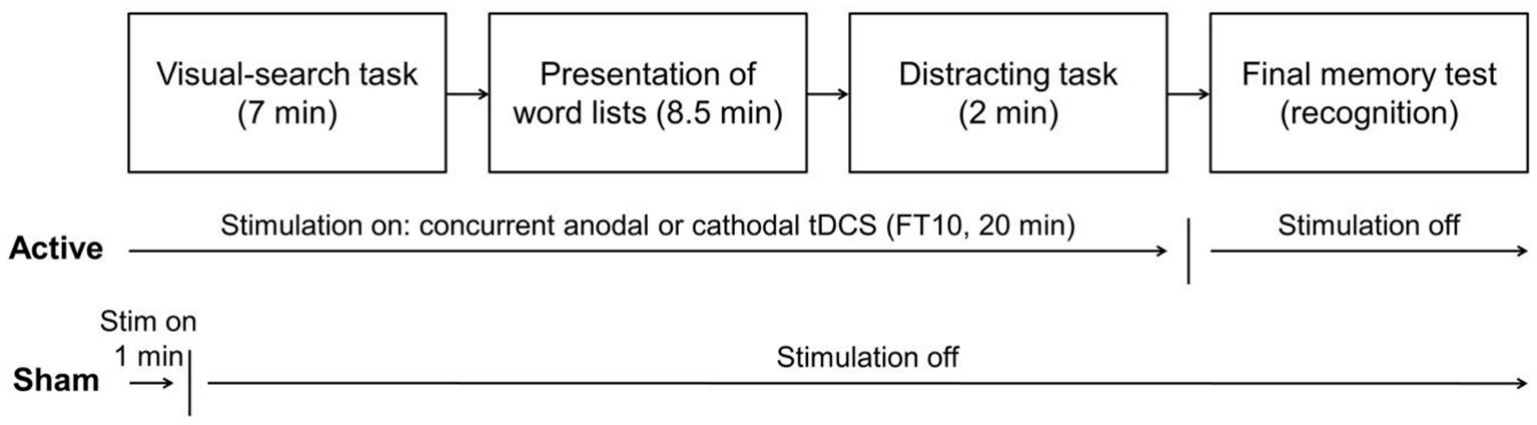
Schematic representation of the experimental procedure.

When the visual-search task ended, the participants received the experimental instructions on a computer screen. These instructions and all subsequent tasks were displayed and controlled by a computer running E-Prime 3.0 software (Psychology Software Tools, 2016). The participants were informed that they would listen to a series of 16 lists of words, and that following the presentation of all the lists they would have to work on a set of arithmetical problems and to perform a final memory test on the words previously presented in the lists. See Figure 1 for a schematic representation of the general procedure.

Following a standard DRM procedure, the words within each list were always presented in the same order, from higher to lower frequency in categorical production or BAS values. Words were presented aurally, one every 2 s. For each participant, a pseudorandom ordering of the 16-list sequence resulted in two subsets of 8 randomly distributed lists (4 categorical and 4 associative), with the last list followed by an on-screen distracting task that lasted 2 min. This task consisted of a series of three-term arithmetical problems presented with a solution that had to be checked for accuracy and required a yes/no response on the computer keyboard. After this task, stimulation was terminated and the participants performed a yes/no recognition memory test. The recognition test included a total of 64 words: the 16 CWs from studied lists (8 associative and 8 categorical), the 8 CWs from non-presented lists (control CWs), 32 studied words (words in position 2 and 7 in the studied lists), and 8 distracting non-presented words (words in position 2 in the non-presented lists). The words in the recognition test were displayed one by one on the center of the computer screen, preceded by a fixation point which lasted 750 ms, and were randomly presented for each participant. The participants were instructed to respond using the keyboard, indicating for each word whether they recognized it from the studied lists (old) or they thought it was a not studied (new) word. If the answer was “yes,” a remember/know judgement was required. The remember/know instructions were adapted from Guillory and Geraci (2010), who took them from Rajaram (1993). After the memory test was completed, all participants completed a questionnaire on tDCS adverse effects (Brunoni et al., 2011). Specifically, they were asked to report the degree to which they experienced a list of side-effects (Headache, Neck pain, Scalp pain, Tingling, Stinging/itching, Burning sensation, Skin redness, Drowsiness, Concentration problems, or Severe mood changes). None of them reported major complaints or discomfort associated with stimulation and, as shown in Table 1, all group means ranged from 1 to 1.92 (i.e., absent to mild). Only skin redness was found to be significantly greater for participants in the Anode and Cathode groups relative to those in the Sham group. The experiment finished with a short debriefing message explaining the experiment and with the request to not reveal details of the session to other students in the participant pool.

**Table 1:** Descriptive statistics (mean and standard deviations) of the responses to the questionnaire on tDCS adverse effects.

	**Anodal**	**Cathodal**	**Sham**	**χ^2^**	^*^ ***p***	**Pairwise comparisons**
Headache	1.28 (0.54)	1.31 (0.68)	1.37 (0.56)	0.76	0.684	
Neck pain	1.32 (0.63)	1.46 (0.81)	1.11 (0.32)	3.46	0.178	
Scalp pain	1.17 (0.48)	1.08 (0.27)	1.00 (0)	3.36	0.187	
Tingling	1.88 (0.80)	1.92 (0.84)	1.89 (0.80)	0.04	0.981	
Stinging/itching	1.68 (0.80)	1.92 (0.84)	1.67 (0.83)	1.81	0.405	
Burning sensation	1.52 (0.77)	1.58 (0.76)	1.41 (0.75)	0.98	0.612	
Reddening of the skin	1.68 (0.63)	1.84 (0.73)	1.11 (0.32)	19.28	<0.001	A > S; C > S
Drowsiness	1.52 (0.77)	1.54 (0.86)	1.37 (0.62)	0.47	0.791	
Concentration problems	1.68 (0.85)	1.61 (0.70)	1.70 (0.67)	0.34	0.842	
Severe mood swings	1.04 (0.20)	1.15 (0.46)	1.00 (0.00)	3.71	0.156	

*Participants rated the side-effects on a scale ranging from 1 to 4 (1: absent; 2: mild; 3: moderate; 4: severe).*

* *p-values from Kruskal-Wallis tests and Dwass-Steel-Critchlow-Fligner pairwise comparisons for significant effects*.

### Design

A mixed factorial design 3 × 2 × 4 was used, with type of stimulation (anodal, cathodal or sham) as a between factor, and type of list (associative or categorical) and type of word (studied, CW, control CW or distractor) as within factors. The dependent variables were derived from the recognition responses to each type of word and from the remember/know judgments.

### Data Analysis

Data on hit rates and critical false alarm rates were analyzed with mixed design repeated-measures ANOVAs, using ${\text{η}}_{\text{p}}^{2}$ as the effect size measure and reporting the corresponding 90% confidence intervals. *Post-hoc* comparisons were performed by way of Tukey's Honest Significant Difference tests. The standard ANOVAs were complemented with Bayesian repeated measures ANOVA analyses. Default priors were used with equal assignment of prior model probability across all models.

Non-parametric signal detection theory measures were used because of the impossibility to test parametric assumptions with yes/no recognition tasks, especially the equality of the signal and the noise standard deviations, and because in DRM tasks it is very common that some subjects have hit or false alarm rates of 1 or 0 (Donaldson, 1996; Stanislaw and Todorov, 1999). The formulas proposed by Zhang and Mueller (2005) were used to calculate non-parametric sensitivity (*A*) and bias (*b*). Since *b* = 1 denotes absence of bias, a logarithmic transformation was applied to convert the variable into a symmetrical one, with a value of 0 denoting absence of bias and negative values denoting liberal criteria and positive values denoting conservative criteria.

All statistical analysis was performed using R Statistical Software (version 4.0.2; R Core Team, 2020) and *jamovi* computer software (The jamovi project, 2021).

## Results

Table 2 shows recognition rates for all the experimental conditions and word types. Overall, across conditions, a strong false recognition effect was evidenced by the high recognition rates of CWs in comparison to those of other non-presented distractor words.

**Table 2:** Mean recognition results (standard deviation) as a function of type of list and type of stimulation.

	**Associative**			**Categorical**		
	**Anodal**	**Cathodal**	**Sham**	**Anodal**	**Cathodal**	**Sham**
**Studied words**						
True recognition	0.66 (0.18)	0.72 (0.12)	0.68 (0.17)	0.77 (0.13)	0.78 (0.14)	0.75(0.13)
Sensitivity (*A*)	0.88 (0.10)	0.90 (0.07)	0.88 (0.09)	0.91 (0.06)	0.89 (0.13)	0.89 (0.10)
Bias [log(*b*)]	0.63 (0.60)	0.54 (0.43)	0.67 (0.37)	0.41(0.46)	0.31 (0.49)	0.51 (0.36)
Remember	0.46 (0.18)	0.46 (0.17)	0.47 (0.19)	0.58 (0.19)	0.53 (0.18)	0.51 (0.18)
Know	0.19 (0.15)	0.26 (0.16)	0.21 (0.14)	0.19 (0.17)	0.25 (0.11)	0.24 (0.15)
**Critical words**						
False recognition	0.53 (0.24)	0.42 (0.33)	0.53 (0.23)	0.57 (0.25)	0.54 (0.24)	0.56 (0.20)
Sensitivity (*A*)	0.78 (0.15)	0.76 (0.16)	0.82 (0.12)	0.84 (0.10)	0.81 (0.14)	0.84(0.12)
Bias [log(*b*)]	0.59 (0.63)	0.73 (0.78)	0.76 (0.55)	0.65 (0.65)	0.70 (0.62)	0.74 (0.44)
Remember	0.32 (0.21)	0.19 (0.20)	0.26 (0.13)	0.28 (0.21)	0.22(0.19)	0.26(0.18)
Know	0.19 (0.15)	0.23 (0.18)	0.27 (0.20)	0.28(0.21)	0.31 (0.18)	0.29 (0.25)
**Distractors**						
False alarms	0.08 (0.19)	0.07 (0.13)	0.06 (0.11)	0.07 (0.11)	0.11 (0.19)	0.07 (0.13)
Critical control	0.15 (0.16)	0.15 (0.20)	0.09 (0.12)	0.11 (0.18)	0.12 (0.23)	0.07 (0.11)

### True Recognition

**Figure 2:**
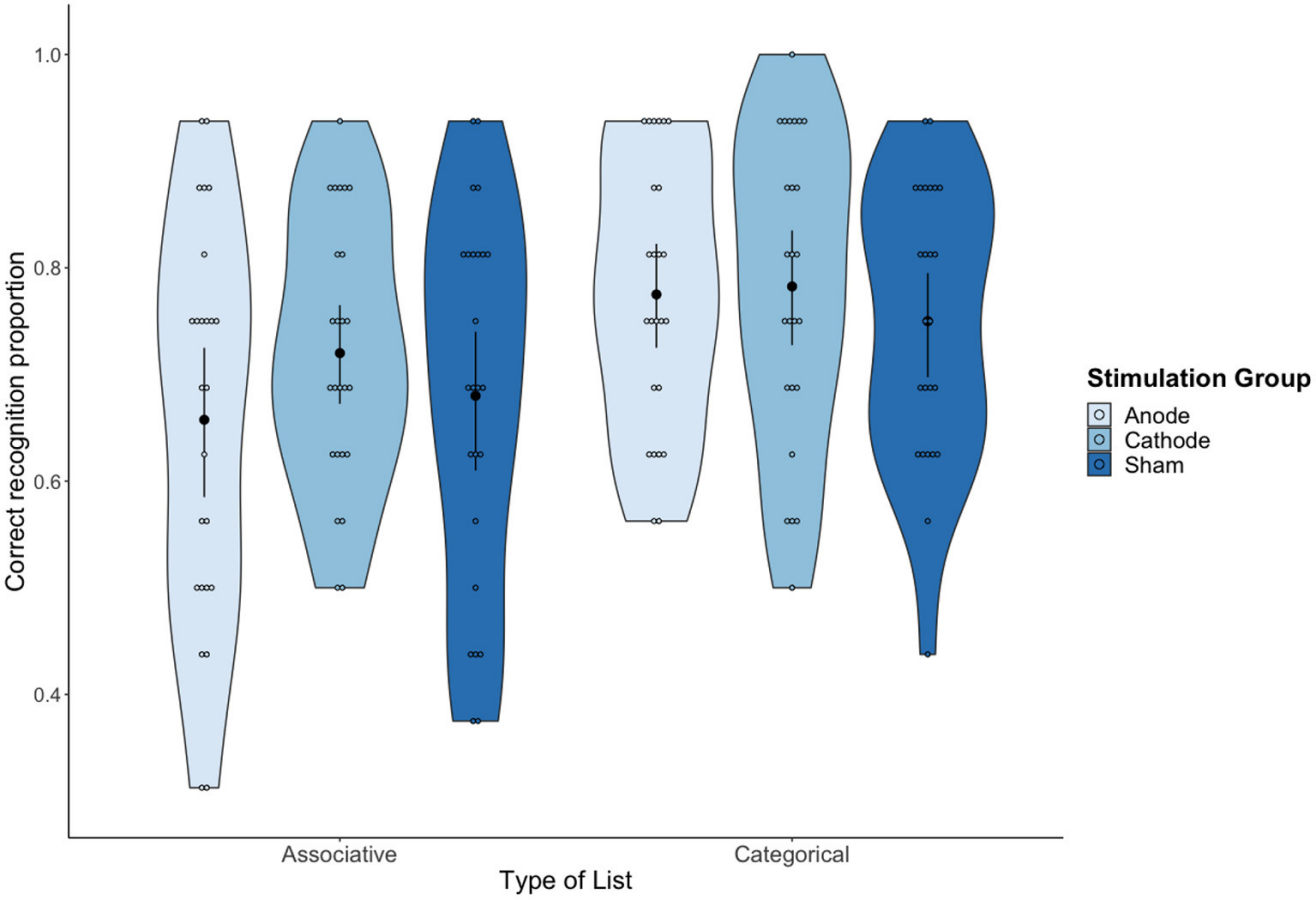
Mean proportion of correct recognition as a function of tDCS condition and Type of list. Error bars represent 95% confidence intervals (CIs).

Data on hit rates (i.e., “yes” responses to studied words) were analyzed with a mixed design repeated-measures ANOVA, with tDCS (Anodal vs. Cathodal vs. Sham) as a between participants variable and with type of list (Associative vs. Categorical) as a within participants variable. There was a significant main effect of type of list, *F*_(1,72)_ = 20.54, *MSE* = 0.01 *p* < 0.001, ${\text{η}}_{\text{p}}^{2}$ = 0.22, 90% CI (0.09, 0.35). On average, the proportion of correct recognition was higher for categorical lists (*M* = 0.77; *SD* = 0.13) than for associative lists (*M* = 0.68; *SD* = 0.16). No other effects were statistically significant (both tDCS condition and interaction effects with *F* < 1), showing that the type of list effect was not modulated by tDCS (see Figure 2).

The standard ANOVA was complemented with Bayesian repeated measures ANOVA analyses, conducted with *jamovi* computer software (The jamovi project, 2021). Default priors were used with equal assignment of prior model probability across all models. The results showed extreme evidence for the type of list effect (BF_10_ = 922.462), and substantial evidence for H_0_ compared to H_1_ in the stimulation condition (BF_01_ = 5.25) and interaction effects (BF_01_ = 4.44).

The 3 × 2 ANOVA on *A* sensitivity scores did not reveal a significant effect of stimulation condition (*F* < 1; BF_01_ = 8.60), type of list, *F*_(1,72)_ = 1.01; *p* = 0.32; BF_01_ = 3.42, or interaction, *F*_(2,72)_ < 1; BF_01_ = 4.17. The analysis on log(b) revealed a statistically significant effect of type of list, *F*_(1,72)_ = 11.63, *MSE* = 0.14, *p* = 0.001, ${\text{η}}_{\text{p}}^{2}$ = 0.14, 90% CI (0.04, 0.26), BF_10_ = 31.39, with associative lists (*M* = 0.62) showing a higher conservative response bias [log(b)] than categorical lists (*M* = 0.41). No other effects reached statistical significance, neither tDCS condition [*F*_(2,72)_ = 1.16, *p* = 0.32; BF_01_ = 3.67 and] nor interaction effects (*F* < 1; BF_01_ = 7.82).

The analysis on Remember/Know responses^2^ revealed a significant effect for “remember” responses in type of list, *F*_(1,72)_ = 12.82, *MSE* = 0.02, *p* = 0.001, ${\text{η}}_{\text{p}}^{2}$ = 0.15, 90% CI (0.04, 0.27), BF_10_ = 40.96, with categorical lists (*M* = 0.54) showing more “remember” responses than associative lists (*M* = 0.46). No significant effects were observed for stimulation condition (*F* < 1, BF_01_ = 5.87) or for the interaction [*F*_(2,72)_ = 1.12; *p* = 0.33, BF_01_ = 4.45).

For “know” responses to true recognized words no significant effects were observed as a function of stimulation condition, *F*_(2,72)_ = 1.6; *p* = 0.19, BF_01_ = 6.39, type of list (*F* < 1, BF_01_ = 0.38) or the interaction (*F* < 1, BF_01_ = 4.87).

### False Recognition

**Figure 3:**
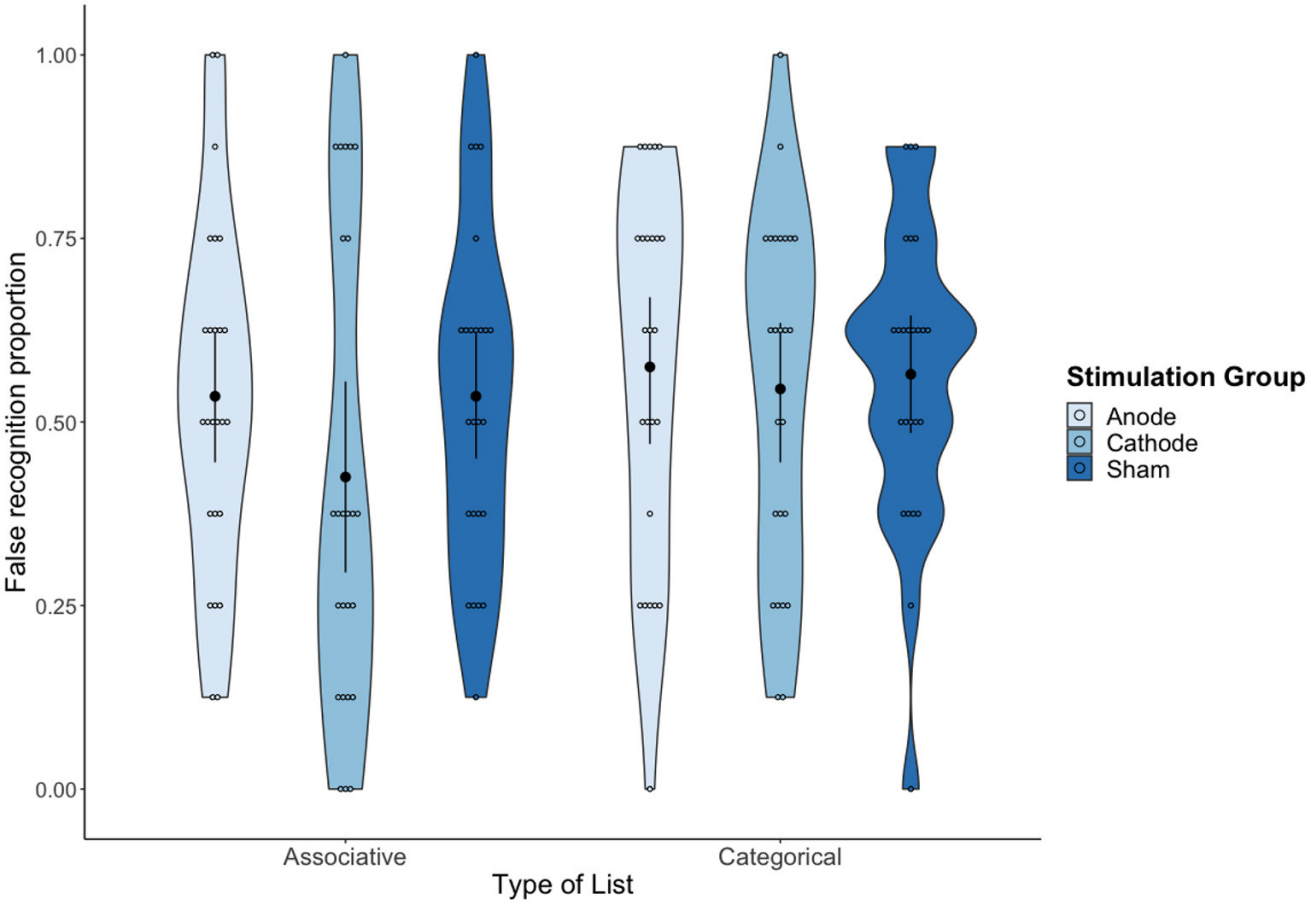
Mean proportion of false recognition as a function of tDCS condition and Type of list. Error bars represent 95% confidence intervals (CIs).

A 3 (tDCS condition: Anodal vs. Cathodal vs. Sham) × 2 (type of list: Associative vs. Categorical) mixed ANOVA on the false recognition rates (i.e., “yes” responses to CWs) showed a statistically significant effect of type of list, *F*_(1,72)_ = 4.64, *MSE* = 0.03, *p* = 0.035, ${\text{η}}_{\text{p}}^{2}$ = 0.06, 90% CI (0.002, 0.16), BF_10_ = 1.37. On average, false recognition was higher for categorical lists (*M* = 0.56; *SD* = 0.23) than for the associative lists (*M* = 0.50; *SD* = 0.27). No other source of variability reached statistical significance (both tDCS condition and interaction with *F* < 1, BF_01_ = 3.90 and BF_01_ = 4.27, respectively), which provides substantial evidence of the absence of an effect of tDCS over false recognition (see Figure 3).

The sensitivity analyses showed a significant effect of type of list, *F*_(1,72)_ = 4.23, *MSE* = 0.02 *p* = 0.04, ${\text{η}}_{\text{p}}^{2}$ = 0.06, 90% CI (0.0009, 0.16), BF_10_ = 1.42, with categorical lists (*M* = 0.83) showing a higher sensitivity rate (*A*) than associative lists (*M* = 0.79). No significant effects were found for the type of stimulation condition, *F*_(2,72)_ = 1.19, *MSE* = 0.03, *p* = 0.31, ${\text{η}}_{\text{p}}^{2}=$ 0.03, BF_01_ = 4.48, or the interaction (*F* < 1, BF_01_ = 6.57). The analyses on *log(b)* did not reveal any statistically significant effect (*F* < 1, BF_01_ values of 5.71 for list type, 4.38 for stimulation and 7.62 for the interaction).

Finally, the proportion of Remember/Know judgments were calculated for false memories. There were no significant effects observed in “remember” responses for type of list (*F* < 1, BF_01_ = 5.67), type of stimulation, *F*_(2,72)_ = 2.47, *p* = 0.09, BF_01_ = 1.42, or interaction (*F* < 1, BF_01_ = 6.19). A significant effect was observed in “know” responses as a function of type of list, *F*_(1,72)_ = 5.87, MSE = 0.03, *p* = 0.018, ${\text{η}}_{\text{p}}^{2}$ = 0.075, 90% CI (0.0071, 0.18), BF_10_ = 2.58. On average, the categorical lists showed more know responses (*M* = 0.29) than associative lists (*M* = 0.23). No significant effects were found in know responses for the stimulation condition (*F* < 1, BF_01_ = 6.43) or the interaction (*F* < 1, BF_01_ = 5.22).

## Discussion

The present experiment aimed to examine the extent to which the right ATL played the same role as the left ATL in semantic processing leading to the generation of false memories; more specifically, it employed a standard DRM paradigm and the application of tDCS to examine the involvement of the right ATL in the production of verbal false memories after studying word lists that, either associatively or categorically, were semantically related to unpresented critical words. On the basis of previous findings by Díez et al. (2017), it was predicted that if the ATL of both hemispheres contributed similarly to semantic processing, a decrease in false recognition in associative lists would be expected following stimulation of the right ATL.

Consistently with previous findings in the literature (Boggio et al., 2009; Gallate et al., 2009; Díez et al., 2017), true recognition was higher for categorical than for associative lists, and was unaffected by ATL stimulation. More relevant for the goal of the experiment, modulating neural activity of the right ATL using tDCS did not modify the elicitation of false memories, with Bayesian analyses showing substantial evidence in favor of the null hypothesis. Neither cathodal nor anodal stimulation altered the rates of false recognition as compared to sham stimulation, and that was true for both associative and categorical lists. A higher overall error rate for categorical lists was the only significant result, small in magnitude, and at odds with most of the reported findings in the false memory literature, with the exception of experiments in which there is a feature or thematic overlap between studied items and critical words that adds to existing associative links (e.g., Coane et al., 2016). In sum, tDCS of the right ATL failed to show effects that were comparable to previously demonstrated effects when the left ATL was stimulated. Pending replication and extension, this finding provides preliminary evidence for an asymmetrical view of the role of the temporal cortex in semantic processing when it comes to producing semantic memory illusions with the DRM procedure.

Studies focusing on how false memory production is affected by altered brain function (either as a consequence of brain damage or as the result of non-invasive stimulation in healthy participants) are not abundant, and even more scarce are studies focusing on the potential role of the ATL in the modulation of memory distortions. And with regard to the specific manipulation aimed at selectively modulating the activity of the right ATL with non-invasive brain stimulations techniques, the present study is, to the best of our knowledge, the first and only one. Still, the lack of evidence for hemispheric symmetry in the pattern of results is in line with recent findings in other related studies employing different methods. Thus, in a meta-analysis of fMRI studies of false memories, Kurkela and Dennis (2016) concluded that the involvement of the temporal cortex in the kind of semantic encoding that leads to false memory was lateralized to the left. More recent evidence for this kind of differential involvement of the two ATLs in verbal and non-verbal semantic processing has been provided by Woollams and Patterson (2018), working with a large sample of semantic dementia patients with lateralized lesions, and by Rice et al. (2018) in their study of a group of postsurgical temporal lobe epilepsy patients, with either left or right anterior temporal lobectomy. In sum, and regardless of which ultimately be the most likely explanation, the data from several years of studies of patients with compromised ATLs are consistent with the idea that the left ATL is prevalently involved in verbal aspects of conceptual processing, while the right ATL is more implicated in pictorial or non-verbal aspects (Gainotti, 2020).

It is also worth noting that the phenomenological experience of participants, as reflected in their remember/know judgments to true and false recognition responses, was not affected by tDCS, echoing prior null findings with stimulation of the parietal cortex (Pergolizzi and Chua, 2015). This finding suggests that the right ATL is not involved in the evaluative processes accompanying recognition decisions for the studied materials. While further systematic analyses are obviously needed, it is interesting that the left ATL has been reported to be involved in the familiarity judgments for verbal materials (e.g., Köhler and Martin, 2020). Given that in the present experiment (as in most other published reports with the DRM procedure) familiarity is as frequently involved as recollection in the production of false memories, additional evidence for the lateralization of familiarity, with a variety of procedures and materials, has the potential to contribute to a better understanding of recognition mechanisms in general and false recognition in particular.

Although the absence of stimulation-related modulation of false memories in the present study can be interpreted in terms of interhemispheric functional differences in the production of semantic memory illusions, other interpretations are also possible. The effects of tDCS on declarative memory are still poorly understood and difficult to replicate at times, most likely due to the multiple factors contributing to them (i.e., stimulation parameters, electrode montages, basal state-dependent neuromodulation, materials to be memorized). A recent meta-analysis of the effects of tDCS on episodic memory revealed that some moderator variables should be considered (Galli et al., 2019). Thus, for example, recall tasks seem to be more sensitive to anodal tDCS than recognition tasks (with cathodal tDCS the tendency seems to be the opposite), especially when associative memory is involved (i.e., Fiori et al., 2011; Flöel et al., 2012; Matzen et al., 2015). In addition, stimulating frontal regions tends to produce larger effects than the stimulation of temporal areas (Galli et al., 2019). Hence, even when the present study embraced a tDCS protocol that proved to be effective at modulating false recognition with the left ATL as the target area (Díez et al., 2017), it could be entirely possible that this protocol (i.e., the intensity of the electric current that is necessary to change the response threshold of the stimulated neurons) is not suitable to change neural activity in the right ATL. The same asymmetries (in anatomy and connectivity; Barrick et al., 2007) potentially producing differentiable functions between the two temporal lobes (left medial temporal regions involved in processing of verbal/ information vs. right homologous regions specially recruited during visual/ processing; Dalton et al., 2016) could also give rise to differences in neuromodulation effectivity. Moreover, it could even be the case that having both ATLs similar functional properties regarding semantic processing, the ability of tDCS to modify their functionality could be different in both hemispheres. Related to this, a recent HD-tDCS study found that stimulating BA22 in the right hemisphere (a site that is slightly more posterior than the target area in our study: BA38/20) modulated insight problem-solving (thought to require semantic integration) relative to sham and left frontopolar stimulation (Salvi et al., 2020). Hence, we recognize that an electrode montage different to the one used here should be considered in future studies. In addition, because false memory effects with DRM procedures are also robust when performance is assessed using recall tasks, future attempts to conceptually replicate the null effect of tDCS over the right ATL to modulate the production of semantic memory illusions should also consider memory tests of this kind that could be more sensitive to external modulations of neural activity.

Beyond the evidence on the asymmetrical involvement of the temporal cortex in semantic processing tasks, the results of the present experiment make a contribution to the quest for the neural correlates of activation and/or gist-formation processes (Roediger et al., 2001; Brainerd and Reyna, 2005) that result in false memory formation. And, when taken together with the findings that reveal a role for the left ATL in false recognition (e.g., Díez et al., 2017), constitute relevant evidence for the assumption that verbal false memories are, to a large extent, a consequence of higher-order semantic processing in the left lateral cortex. They also offer support to explanations of memory distortions by neuroscience-based semantic approaches that, like the hub-and-spoke model (Patterson and Lambon Ralph, 2016; Lambon Ralph et al., 2017), open the door to assume that such errors are critically related to integrative, conceptual processes taking place in the ATL. At the same time, these results point to the need for such models to be further expanded and replicated with independent samples, more inclusive in terms of characteristics such as gender or handedness, and to pay closer attention to the specifics of the particular brain areas involved in the different processes and subprocesses considering the combined use of NIBS and neuroimaging techniques.

## Data Availability Statement

The raw data supporting the conclusions of this article will be made available by the authors, without undue reservation.

## Ethics Statement

The studies involving human participants were reviewed and approved by CEIBA - Comité de Ética de la Investigación y Bienestar Animal (CEIBA2017-0265). The patients/participants provided their written informed consent to participate in this study.

## Author Contributions

MA collected the data. ED analyzed the data. All authors contributed to the design of the experiment and contributed to writing the manuscript.

## Funding

The work reported here was partially supported by the Spanish Ministry of Economy, Industry and Competitiveness (Grant PSI2017-82748-P), and by Junta de Castilla y León (Grant SA052G18).

## Conflict of Interest

The authors declare that the research was conducted in the absence of any commercial or financial relationships that could be construed as a potential conflict of interest.

## Publisher's Note

All claims expressed in this article are solely those of the authors and do not necessarily represent those of their affiliated organizations, or those of the publisher, the editors and the reviewers. Any product that may be evaluated in this article, or claim that may be made by its manufacturer, is not guaranteed or endorsed by the publisher.

## Footnotes

^1^A non-parametric measure of response bias was calculated for each subject, and all the data from participants scoring above or below 1.96 SDs from the average bias in the total sample were excluded from the analyses. Following Díez et al. (2017), this screening procedure was aimed at excluding participants with extreme response patterns, as evidenced in their recognition responses (hit rates and false alarms) to the items.

^2^Remember/know scores were calculated as a proportion of the total possible responses in each condition. Analyses were also performed by calculating them as a proportion of the “yes” responses. These analyses showed no significant effects of tDCS, list type or interaction for either correct or false recognition.
